# Genetic Addiction Risk Severity Assessment Identifies Polymorphic Reward Genes as Antecedents to Reward Deficiency Syndrome (RDS) Hypodopaminergia’s Effect on Addictive and Non-Addictive Behaviors in a Nuclear Family

**DOI:** 10.3390/jpm12111864

**Published:** 2022-11-08

**Authors:** Catherine A. Dennen, Kenneth Blum, Abdalla Bowirrat, Panayotis K. Thanos, Igor Elman, Mauro Ceccanti, Rajendra D. Badgaiyan, Thomas McLaughlin, Ashim Gupta, Anish Bajaj, David Baron, B. William Downs, Debasis Bagchi, Mark S. Gold

**Affiliations:** 1 Department of Family Medicine, Jefferson Health Northeast, Philadelphia, PA 08033, USA; 2 Division of Addiction Research & Education, Center for Sports and Mental Health, Western University of Health Sciences, Pomona, CA 91766, USA; 3 Division of Nutrigenomics, The Kenneth Blum Behavioral & Neurogenetic Institute, Austin, TX 78701, USA; 4 Institute of Psychology, ELTE Eötvös Loránd University, Egyetem tér 1–3, 1053 Budapest, Hungary; 5 Department of Psychiatry, School of Medicine, University of Vermont, Burlington, VT 05405, USA; 6 Department of Psychiatry, Wright State University Boonshoft School of Medicine and Dayton VA Medical Centre, Dayton, OH 45324, USA; 7 Center for Genomics and Applied Gene Technology, Institute of Integrative Omics and Applied Biotechnology (IIOAB), Nonakuri, Purba Medinipur 721172, West Bengal, India; 8 Department of Molecular Biology and Adelson School of Medicine, Ariel University, Ariel 40700, Israel; 9 Behavioral Neuropharmacology and Neuroimaging Laboratory, Clinical Research Institute on Addictions, Department of Pharmacology and Toxicology, Jacobs School of Medicine and Biomedical Sciences, University at Buffalo, Buffalo, NY 14203, USA; 10 Center for Pain and the Brain (P.A.I.N Group), Department of Anesthesiology, Critical Care & Pain Medicine, Boston Children’s Hospital, Boston, MA 02115, USA; 11 Società Italiana per il Trattamento dell’Alcolismo e le sue Complicanze (SITAC), ASL Roma, Sapienza University of Rome, 00185 Rome, Italy; 12 Department of Psychiatry, South Texas Veteran Health Care System, Audie L. Murphy Memorial VA Hospital, Long School of Medicine, University of Texas Medical Center, San Antonio, TX 78229, USA; 13 Reward Deficiency Clinics of America, Austin, TX 78701, USA; 14 Future Biologics, Lawrenceville, GA 30043, USA; 15 Bajaj Chiropractic Clinic, New York, NY 10010, USA; 16 Department of Pharmaceutical Sciences, Southern University College of Pharmacy, Houston, TX 77004, USA; 17 Department of Psychiatry, Washington University School of Medicine, St. Louis, MO 63110, USA

**Keywords:** genetic addiction risk severity (GARS), hypodopaminergia, polymorphisms, reward circuitry, reward deficiency syndrome (RDS), single nucleotide polymorphisms (SNPs)

## Abstract

This case series presents the novel genetic addiction risk score (GARS), which shows a high prevalence of polymorphic risk alleles of reward genes in a nuclear family with multiple reward deficiency syndrome (RDS) behavioral issues expressing a hypodopaminergic antecedent. The family consists of a mother, father, son, and daughter. The mother experienced issues with focus, memory, anger, and amotivational syndrome. The father experienced weight issues and depression. The son experienced heavy drinking, along with some drug abuse and anxiety. The daughter experienced depression, lethargy, brain fog, focus issues, and anxiety, among others. A major clinical outcome of the results presented to the family members helped reduce personal guilt and augment potential hope for future healing. Our laboratory’s prior research established that carriers of four or more alleles measured by GARS (*DRD1-DRD4*, *DAT1*, *MOR*, *GABABR3*, *COMT*, *MAOAA*, and *5HTLPR*) are predictive of the addiction severity index (ASI) for drug abuse, and carriers of seven or more alleles are predictive of severe alcoholism. This generational case series shows the impact that genetic information has on reducing stigma and guilt in a nuclear family struggling with RDS behaviors. The futuristic plan is to introduce an appropriate DNA-guided “pro-dopamine regulator” into the recovery and enhancement of life.

## 1. Introduction

In an attempt to understand reward dependence behaviors and associated neurogenetic impairments leading to brain reward dysphoria or depression and thus social ineptness, as well as drug and non-drug seeking addictive behaviors, more in-depth research is required. Our laboratory coined the term “reward deficiency syndrome” (RDS) in 1996 to describe a standard genetic rubric of impulsive, compulsive, and addictive behaviors, which is an important emerging psycho-neuro-genetic concept now being adopted worldwide, and it is listed as a disorder in the SAGE 2017 Encyclopedia of Abnormal Psychology [1]. To highlight the importance of this topic, using the search term “reward deficiency”, there are 1458 articles listed in PubMed (as of 28 August 2022), and using the search term “reward deficiency syndrome”, there are 224 articles, of which 47% or so are independent of our laboratory. Interestingly, out of these research reports, roughly 20% included the comorbidity of depression and many related RDS behaviors, including substance use disorder (SUD), attention deficit hyperactivity disorder (ADHD), obsessive-compulsive disorder (OCD), and anxiety, among others. It is important to note that the Diagnostic and Statistical Manual of Mental Disorders, Fifth Edition (DSM-5) compartmentalizes various behaviors that may not actually reflect brain dysfunction. This superstition has been reflected in reports by Hyman’s group, which suggest that future research should focus on the root causes of these psychiatric disorders rather than just trying to identify the symptoms. The therapeutic target should address the etiologic root, such as dopamine dysregulation, rather than any symptom [2]. Certainly, the DSM system is still a mainstream diagnostic tool to obtain psychiatric diagnoses, but there is the potential that RDS become a feature in future editions of the DSM whereby the genetic addiction risk score (GARS) test could be used to identify, for example, even pre-addiction.

It is noteworthy that, in spite of flawed psychiatric genetic research, including large genome-wide association studies (GWAS) regarding the systematic mapping of genes associated with RDS behaviors, we are proposing the genotyping of RDS probands compared to a highly screened control population. In the search for an accurate, gene-based test to identify heritable risk factors for RDS, hundreds of published research studies involving the function of dopamine in addictive behaviors, such as the risk for impulsive/compulsive behavior disorders and drug dependence, were used. A preponderance of evidence observed indicated a common neurobiological mechanism associated with a polymorphic allelic propensity for hypodopaminergia and, in adolescents due to epigenetic factors, hyperdopaminergia. In another paper that we published, it turned out that in adolescence, in spite of being born with a number of polymorphisms in reward genes as denoted by GARS, it was also found that, via developmental phases and possibly epigenetic adaptations, a hyperdopaminergia is observed [3].

Most recently, we published a large meta-analysis involving 74,556 case–control subjects accessed by a literature survey for alcohol use disorder (AUD) [4]. This analysis calculated the Hardy–Weinberg Equilibrium of each polymorphism in cases and controls. If available, the Pearson’s *χ*^2^ test or Fisher’s exact test was applied to comparisons of the gender, genotype, and allele distribution. The statistical analyses found that the 95% CI for OR and the 8% post risk estimation of the population’s alcoholism prevalence revealed a significant detection. The OR results showed significance for the *dopamine receptor D2 (DRD2)*, *dopamine receptor D3 (DRD3)*, *dopamine receptor D4 (DRD4)*, *dopamine transporter (DAT1)*, *catecholamine-methyltransferase (COMT)*, *opioid receptor mu 1 (OPRM1)*, and *serotonin-transporter-linked promoter region (5HTTLPR)* at 5%. In one article by Blum et al., a possible solution was illustrated by comparing the prevalence of the *DRD2 A1* allele in unscreened controls (33.3%) to “super-controls” (highly screened RDS controls (3.3%) in the proband and family) [5]. However, in contrast to one-gene-one disease, RDS is polygenetic and extremely complex. As a cautionary note, we would like to emphasize that any RDS-related behaviors must be removed from the control group in order to achieve the best statistical analysis and to prevent the phenotype from being compared with disease-ridden controls. It is widely known that drugs of abuse (i.e., opioids, etc.) and intrinsic reward may interact with mesolimbic pathways to activate a common mechanism of neural plasticity in the nucleus accumbens (NAc). Blum et al. suggested an endogenous opioid-induced neuroplasticity of dopaminergic neurons in the ventral tegmental area (VTA) that influenced natural and opiate (morphine) rewards [6]. In fact, this was experimentally observed by Pitchers et al. earlier [7].

While Blum’s group published their RDS construct in the mid-1990s as an umbrella for many linked psychiatric behaviors, thousands of studies have since supported the role of genetically or even epigenetically hypodopaminergic behaviors such as alcoholism, heroin, psychostimulant, cannabis, and nicotine dependence, carbohydrate craving, pathological aggression, pathological gambling, sex addiction, high-risk taking behavior, and certain personality disorders, including schizoid avoidance behavior, borderline personality, impaired executive function, inability to cope with stress in the family, posttraumatic stress disorder, and most recently excessive internet video gaming [8,9,10,11,12,13,14,15,16,17,18,19,20,21,22]. For example, in terms of internet addiction and gaming, the results of a mediated moderation analysis by Kim et al. showed that, when experiencing interpersonal stressors, individuals with both the *Taq1 A1* allele and the *C957T T* allele displayed greater problematic gaming scores compared to non-carriers [22]. Additionally, avoidant coping had a significant role in mediating the interaction impact of interpersonal stress and the combined *DRD2* polymorphisms. Moreover, Blum et al. found a strong association between the *DRD2 Taq A1* allele and schizoid/avoidant behavior [23]. Additionally, an albeit weaker association between the 480-bp variable number tandem repeats (VNTR) 10/10 allele of the *DAT1* gene and schizoid/avoidant behavior was similarly found. One plausible mechanism for at least the *DRD2 A1* allele is that homozygote carriers of this polymorphism have 30–40% fewer D2 receptors than *DRD2 A2* allele carriers [24].

The GARS screening test will present a new opportunity to discover mechanisms of psychological characteristics, genetic factors, and causal pathways involved in addictions. Additional scientific evidence, which involves a future meta-analysis of all the available data, is a work in progress.

The literature was reviewed to ascertain each allele and related polymorphism proposed in the GARS panel. This was specifically done for alcoholism, but similar results have also been shown for many reward genes (Table 1).

Genetic risk assessment allows for the early detection of genetic vulnerability to addictive behaviors [25]. Additionally, according to a fairly moderate amount of literature and research, reward gene polymorphisms predispose individuals to an increased risk of all RDS behavior subtypes, including SUD and other disorders such as hoarding [26]. The GARS test has been developed to identify one’s risk potential for these addictive-like behaviors [27].

The objective of this article is to substantiate earlier work by examining a nuclear family ridden with multiple RDS behaviors, including substance and non-substance use, through the utilization of genotyping and the GARS test [28].

**Table 1:** Summary of studies used for controls *.

GENE	Risk Allele	Positive Phenotypes	Studies	Case (N) Control (N)	Meta-Analysis	Sig (*p*)	Ref
*DRD1*	*Rs4532* and specific haplotype rs686*T-rs4532*G within the *DRD1* gene	AUD, aggression, and impulsivity	3	Case (569) Control (218)	NONE	<0.01–0.001	[27]
*DRD2*	*Rs1800497*	Severe alcoholism, long-term drinking, AD, parental rule-setting, comparison severe vs. less severe alcoholics, relapse and ASI after 12 years in 12-step programs, family linkage, heavy drinking, early onset, stress, harm avoidance and antisocial behavior related to AUD, severe medical consequences, mortality hospitalization, COAs, parental history of alcoholism, and drinking in the general population,	62	Case (17,382) Control (17,036)	4	<0.04–0.009	[29]
*DRD3*	*DRD3 Ser9Gly* polymorphism (*rs6280*)	AD, anhedonia, MDD, and obsessive compulsive drinking	3	Case (545) Control (156)	NONE	<0.001–0.008	[27]
*DRD4*	*Rs180095 48bP* repeat VNTR	Risk factor for alcoholism, AD smoking behavior, polysubstance abuse, higher rates of novelty seeking, higher lifetime alcoholism, generalized addiction, increased influence of peer pressure to drink, problematic alcohol use, increase the risk for severity of alcoholism, blunted response to alcohol cues, increase in alcohol craving, increased risk for social bonding with fellow alcoholics.	48	Case (11,740) Control (9365)	2	<0.06–0.005	[17]
*DAT1*	*9R allele* compared to *10R*	Alcoholism, alcohol consumption, AWS, DTs, number of drinking days, vulnerability to alcoholism, and families with alcoholism compared to families without alcoholism	24	Case (4644) Control (3761)	2	<0.05–0.009	[6]
*COMT*	*Rs4680* *COMT Val158Met*	AD, alcohol intake past year, generalized SUD, alcohol and tobacco consumption, drug abuse, in alcoholics reduced dopamine receptor sensitivity	75	Case (10,018) Control (8861)	1	<0.01–0.001	[8]
*OPRM1*	*OPRMI* (*rs1799971*)	AD, severity of AWS, sensitivity to dopamine receptors, alcohol consumption, depression, response to alcohol cues and relapse risk, alcohol sensitivity in adolescents, drinking frequency, vulnerability for alcohol to hijack the reward system, alcohol craving, alcohol-related hospital readmission, more readmissions, and fewer days until the first readmission	15	Case (6428) Control (5196)	1	<0.047–0.006	[12]
*GABRB3*	*Receptor beta3 subunit (GABRB3) 181 variant*	The risk for alcoholism, the onset of drug abuse in COAS, parental transmission and alcoholism, hypodopaminergia, Mood-related alcohol expectancy, drinking refusal self-efficacy, depression, and prevalence in COAS	6	Case (196) Control (0)	NONE	<0.05–0.007	[6]
*MAOA*	*30 BP VNTR-3.5R, 4R DN repeat* polymorphisms	AD, impulsivity, antisocial personality, susceptibility to alcoholism, smoking behavior, poor psychosocial environment, and lower age of onset of alcoholism.	5	Case (731) Control (1111)	NONE	<0.043–0	[5]
*SLC6A4* *(5HTTLPR)*	promoter region (*5HTTLPR*) (*rs25531*)	AD, anxiety, age of onset, cue craving, lower socialization, depression, and poly drug abuse	27	Case (13,328) Control (2982)	2	<0.03–0.001	[9]
TOTAL	NA	NA	268	Case (65,581) Control (48,686)	10	<0.06–0.009	NA

* Table 1: The columns labeled “Studies” and “Meta-analysis” refers to the number of studies and meta-analyses associated with each gene/risk allele respectively. The column labeled “Case (N) Control (N)” refers to the number of cases and controls associated with each gene/risk allele. The column labeled “Sig (*p*)” refers to the statistical significance/*p*-value. The column labeled “reference” refers to the reference number in this paper’s bibliography that is associated with each gene/risk allele. Abbreviations: N (values), dopamine receptor D1 (DRD1), dopamine receptor D2 (DRD2), dopamine receptor D3 (DRD3), dopamine receptor D4 (DRD4), dopamine transporter (DAT1), catecholamine-methyltransferase (COMT), opioid receptor mu 1 (OPRM1), gamma-aminobutyric acid type A receptor subunit beta3 (GABRB3), monoamine oxidase A (MAOA), and serotonin-transporter-linked promoter region (5HTTLPR), alcohol use disorder (AUD), major depressive disorder (MDD), alcohol dependence (AD), children of alcoholics (COAS), alcohol withdrawal symptoms (AWS), and delirium tremens (DTs).

## 2. Materials and Methods

Instrumentation, data collection procedures, and the analytical methodology utilized to attain GARS and successive research objectives have been described elsewhere [29]. It is thought that a GARS-type screening test will present a new opportunity in the identification of causal pathways and related mechanisms involving genetic factors, psychological characteristics, and addictions that are still in need of additional scientific evidence. The GARS test is a direct-to-consumer (DTC), non-diagnostic DNA genetic testing kit. The GARS genetic test determines genetic risk for RDS behaviors quantitatively by enumerating the number of single nucleotide polymorphisms (polymorphic risk alleles) detected. The GARS testing was approved by the IRB committee of Western University Health Sciences to assist in pinpointing the effects of dopamine agonists. Each patient signed an approved informed consent.

### Sample Collection and Processing Utilized to Obtain Data

Buccal cells were collected from each patient utilizing an established minimally invasive collection kit. Samples were collected using Sterile Copan 4N6FLOQ Swabs (regular size tip in 109mm long dry tube with an active drying system). The cells were collected from both cheeks by rubbing the swab on each side of the mouth at least 25 times, and then the swab was returned to the specimen tube. Appropriate controls were used and validated for every stage of sample processing, including known DNA standards and non-template controls.

An index of the genes incorporated in the GARS panel and the specific risk polymorphisms are provided in Table 2, Table 3 and Table 4. Each polymorphism was chosen based on SUD, a subset of RDS, and its known contribution to a state of low dopaminergic or hypodopaminergic functioning in the brain’s reward circuitry. Samples also underwent sex determination utilizing PCR amplification and capillary electrophoresis in order to detect *AMELX* and *AMELY* (*AMELX*’s intron 1 contains a 6 bp deletion relative to intron 1 of *AMELY)*.

DNA was extracted from buccal samples utilizing a Mag-Bind Swab DNA 96 Kit (Custom M6395–01, Omega Bio-Tek, Norcross, GA, USA) and the MagMAX Express-96 Magnetic Particle Processor (Applied Biosystems, Foster City, CA, USA). Extracted DNA was quantified for total human gDNA utilizing the TaqMan RNase P assay (Life Technologies, Carlsbad, CA, USA) on a QuantStudio 12k Flex (Thermo Fisher Scientific, Waltham, MA, USA).

Genetic variation testing was performed utilizing (1) Real-time PCR with TaqMan^®^ allele-specific probes on the QuantStudio 12K Flex or (2) iPlex reagents on the Agena MassARRAY^®^ system, plus (3) Proflex PCR and size separation using the SeqStudio Genetic Analyzer.

Single nucleotide polymorphisms (SNPs) were genotyped (Table 2) utilizing the QuantStudio 12K Flex Real-Time PCR system, and commercially available or custom TaqMan RT-PCR assays (Thermo Fisher Scientific, Waltham, MA, USA) were also utilized (see Table 5 for Assay IDs and context sequences). For every reaction, 2.25 µL of normalized DNA (10 ng total) was mixed with 2.75 µL of the assay master mix, which was then subjected to RT-PCR amplification and detection. Thermal cycling conditions recommended by the manufacturer were utilized, and genotypes were called using TaqMan Genotyper Software v1.3 (Life Technologies, Carlsbad, CA, USA).

The Agena MassARRAY^®^ system was also utilized for single nucleotide polymorphism genotyping, and iPlex reagents were utilized (see Table 3 for iPlex PCR primer sequences). Primers are multiplex, so only one reaction is needed for every sample. For every reaction, 2 uL of normalized DNA (10 ng total) was mixed with the iPlex Pro PCR cocktail. The reaction was amplified on a ProFlex thermocycler utilizing the Agena manufacturer’s recommended PCR conditions. Amplified DNA was then SAP treated, followed by an extension. The iPLEX Extension Reaction Product was then desalted utilizing a dry resin method. Samples were then dispensed onto a 96 well SpectroCHIP Array utilizing the MassARRAY Nanodispenser. Genotypes were called using the MassARRAY Analyzer Software.

For fragment genotyping, two multiplexed PCR reactions (50 µL total volume) were required. Reaction A included 5′ fluorescently labeled primers, forward primers, and non-labeled reverse primers for *AMELOX/Y*, *DAT1*, *MAOA*, and the *GABRB3* dinucleotide repeat (with sets at 150 nM, 120 nM, 120 nM, and 480 nM primer concentrations, respectively). Reaction B included 5′ fluorescently labeled forward primers and non-labeled reverse primers for *DRD4* and the *SLC6A4 HTTLPR*, all in 120 nM concentrations. For all PCR reactions, 2 ng of DNA was amplified with primers, 25 µL OneTaq HotStart MasterMix (New England Biolabs, Ipswich, MA, USA), and water. For reaction B, 5 µM 7-deaza-dGTP (Thermo Fisher Scientific, Waltham, MA, USA) was added to the above recipe. Primer details are listed in Table 6.

Amplifications were performed utilizing the touchdown PCR method. An initial 95 °C incubation for 10 min was followed by two cycles of 95 °C for 30 s, 65 °C for 30 s, and 72 °C for 60 s. The annealing temperature was decreased every two cycles from 65 °C to 55 °C in 2 °C increments (10 cycles total), followed by 30 cycles of 95 °C for 30 s, 55 °C for 30 s, and 72 °C for 60 s, and a final 30-min incubation at 60 °C, then held at 4 °C. A 10 µL aliquot of the reaction B amplicon was further subjected to MspI restriction digest (37 °C for 1h) to interrogate rs25531 (with 1 unit of restriction enzyme and 1X Tango Buffer, Thermo Fisher Scientific, Waltham, MA, USA).

Capillary electrophoresis was utilized for fragment detection. Reactions 1 and 2 were mixed in a 2:1 ratio, and 1 µL of this amplicon mixture was added to 9.5 µL mixed LIZ1200 size standard/-formamide (Thermo Fisher Scientific, Waltham, MA, USA recommended concentrations). For the detection of rs25531, 1 uL of restriction digest mixture was added to 9.5 µL of LIZ1200 ± formamide. Both mixtures underwent capillary electrophoresis on the SeqStudio (run time 60 min, voltage 5000 V, 10s injection at 1200 V) and were analyzed with GeneMapper 5 software (Life Technologies, Carlsbad, CA, USA).

The patients in this case received a personalized report outlining the results. The report contains a GARS Score (based on a scale of 1–22), which provides the sum of all risk alleles for that particular individual. Additionally, the results for each individual are categorized as high, moderate, or low-risk behavior frequency for various substance and non-substance behaviors. The reports are created to assist users in comprehending the meaning of their results and the appropriate next steps.

The genetic panel was specifically chosen based on polymorphisms of several reward genes that have been linked to chronic dopamine deficiency and drug-related reward-seeking behavior (Table 5). In Table 5, we display the current polymorphic risk alleles of the GARS panel.

PubMed provides frequency data for major and minor alleles but not for population prevalence. SNPedia provides population diversity percentages for homozygous SNP, homozygous normal, and heterozygous for all except one of our SNPs in the following populations (*rs4532*, *rs1800497*, *rs6280*, *rs1800955*, *rs4680*, and *rs1799971*) expressed in Table 6. Unfortunately, there are currently no data on the population prevalence of VNTRs or dinucleotide repeats (Table 2, Table 3, Table 4, Table 5, Table 6, Table 7 and Table 8).

## 3. Results

The four family members (mother, father, daughter, and son) were genotyped, and their respective DNA was processed according to the GARS test as described above. The interpretation of these results was explained to each patient by one of us (KB). Specifically, the mother displayed ten risk alleles (Table 9); the father displayed nine risk alleles (Table 10); the son displayed eleven risk alleles (Table 11); and the daughter also displayed eleven risk alleles (Table 12). While we received signed informed consent for this case series, we have deidentified the patient by using their accession number instead of their initials or actual name for privacy purposes. The mother (135530) is a 52-year-old Asian female; the father (1355511) is a 56-year-old Caucasian male; the son (184700) is a 21-year-old Asian/Caucasian male; and the daughter is a 19-year-old Asian/Caucasian female (142430) The respective GARS results for each proband are displayed in Table 9, Table 10, Table 11 and Table 12.

### 3.1. Mother (135530)

**Table 9:** Mother’s GARS test results *.

Gene	Identifiers	Risk Allele	Patient Results Risk	Risk Allele Count
*DRD1*	*rs4532*	A	A/A	2
*DRD2*	*rs1800497*	A	A/G	1
*DRD3*	*rs6280*	C	C/T	1
*DRD4*	*rs1800955*	C	C/T	1
*OPRM1*	*rs1799971*	G	A/G	1
*COMT*	*rs4680*	G	A/A	0
*DAT1*	*rs28363170*	<9 Repeats	10/10R	0
*5HTTLPR*	*rs4795541*	S, LG	S/S	2
*MAOA*	*rs768062321*	3.5 R, 4R	4R/4R	2
*GABRB3*	*rs764926719*	181	185/185	0
*DRD4*	*rs761010487*	≥7 Repeats	2R/4R	0

* Abbreviations: Dopamine receptor D1 (*DRD1*), dopamine receptor D2 (*DRD2*), dopamine receptor D3 (*DRD3*), dopamine receptor D4 (*DRD4*), opioid receptor mu 1 (*OPRM1*), catecholamine-methyltransferase (*COMT*), dopamine transporter (*DAT1*), and serotonin-transporter-linked promoter region (*5HTTLPR*), monoamine oxidase A (*MAOA*), gamma-aminobutyric acid type A receptor subunit beta3 (*GABRB3*).

### 3.2. Father (135511)

**Table 10:** Father’s GARS test results *.

Gene	Identifiers	Risk Allele	Patient Results Risk	Risk Allele Count
*DRD1*	*rs4532*	A	A/A	2
*DRD2*	*rs1800497*	A	G/G	0
*DRD3*	*rs6280*	C	C/T	1
*DRD4*	*rs1800955*	C	C/T	1
*OPRM1*	*rs1799971*	G	A/A	0
*COMT*	*rs4680*	G	A/A	0
*DAT1*	*rs28363170*	<9 Repeats	10/10R	0
*5HTTLPR*	*rs4795541*	S, LG	S/LA	1
*MAOA*	*rs768062321*	3.5 R, 4R	3R	0
*GABRB3*	*rs764926719*	181	181/181	2
*DRD4*	*rs761010487*	≥7 Repeats	7R/7R	2

* Abbreviations: Dopamine receptor D1 (*DRD1*), dopamine receptor D2 (*DRD2*), dopamine receptor D3 (*DRD3*), dopamine receptor D4 (*DRD4*), opioid receptor mu 1 (*OPRM1*), catecholamine-methyltransferase (*COMT*), dopamine transporter (*DAT1*), and serotonin-transporter-linked promoter region (*5HTTLPR*), monoamine oxidase A (*MAOA*), gamma-aminobutyric acid type A receptor subunit beta3 (*GABRB3*).

### 3.3. Son (184700)

**Table 11:** Son’s GARS test results *.

Gene	Identifiers	Risk Allele	Patient Results Risk	Risk Allele Count
*DRD1*	*rs4532*	A	A/A	2
*DRD2*	*rs1800497*	A	A/G	1
*DRD3*	*rs6280*	C	C/T	1
*DRD4*	*rs1800955*	C	C/T	1
*OPRM1*	*rs1799971*	G	A/G	1
*COMT*	*rs4680*	G	A/A	0
*DAT1*	*rs28363170*	<9 Repeats	10/10R	0
*5HTTLPR*	*rs4795541*	S, LG	S/S	2
*MAOA*	*rs768062321*	3.5R, 4R	3R/4R	1
*GABRB3*	*rs764926719*	181	181/185	1
*DRD4*	*rs761010487*	≥7 Repeats	4R/7R	1

* Abbreviations: Dopamine receptor D1 (*DRD1*), dopamine receptor D2 (*DRD2*), dopamine receptor D3 (*DRD3*), dopamine receptor D4 (*DRD4*), opioid receptor mu 1 (*OPRM1*), catecholamine-methyltransferase (*COMT*), dopamine transporter (DAT1), and serotonin-transporter-linked promoter region (*5HTTLPR*), monoamine oxidase A (*MAOA*), gamma-aminobutyric acid type A receptor subunit beta3 (*GABRB3*).

### 3.4. Daughter (142430)

**Table 12:** Daughter’s GARS test results *.

Gene	Identifiers	Risk Allele	Patient Results Risk	Risk Allele Count
*DRD1*	rs4532	A	A/A	2
*DRD2*	rs1800497	A	A/G	1
*DRD3*	rs6280	C	C/T	1
*DRD4*	rs1800955	C	C/T	1
*OPRM1*	rs1799971	G	A/G	1
*COMT*	rs4680	G	A/A	0
*DAT1*	rs28363170	<9 Repeats	10/10R	0
*5HTTLPR*	rs4795541	S, LG	S/S	2
*MAOA*	rs768062321	3.5R, 4R	3R/4R	1
*GABRB3*	rs764926719	181	181/185	1
*DRD4*	rs761010487	≥7 Repeats	4R/7R	1

* Abbreviations: Dopamine receptor D1 (*DRD1*), dopamine receptor D2 (*DRD2*), dopamine receptor D3 (*DRD3*), dopamine receptor D4 (*DRD4*), opioid receptor mu 1 (*OPRM1*), catecholamine-methyltransferase (*COMT*), dopamine transporter (*DAT1*), and serotonin-transporter-linked promoter region (*5HTTLPR*), monoamine oxidase A (*MAOA*), gamma-aminobutyric acid type A receptor subunit beta3 (*GABRB3*).

In addition, post-psychiatric reviews of each family member and previous psychiatric diagnoses are presented in Table 13. It is noteworthy that there is also evidence for multiple RDS behaviors in this family’s extended family. For example, The Father’s two brothers are both over 300 pounds. The mother’s brother currently misuses alcohol and possibly other drugs, has been diagnosed with ADHD, is a risk-taker (i.e., lost his money), and is verbally abusive. A nephew, at the age of 16, has been in juvenile court for drug charges and is known to be violent (smashed TV, etc.). Genetic testing has been restricted to only the nuclear family at this time.

## 4. Discussion

It is of interest that the current family displays similar RDS behaviors to what we published earlier in terms of displaying a high prevalence of dopaminergic gene polymorphisms (*DRD2*) in a nuclear and extended family ridden with multiple RDS addictive behaviors [30]. In the previous family study, among the genotyped family members, the *DRD2 Taq1* A1 allele was significantly more often found in the RDS families vs. controls. The *TaqA1* allele occurred in 100% of Family A individuals (N = 32) and 47.8% of Family B subjects (N = 23).

Published research demonstrates how the GARS test can be utilized to identify specific neurotransmitter pathways that are at risk for a signal breakdown in the brain’s reward circuitry. While having this genetic knowledge does not per se address therapeutic or tertiary treatment directly, it has important clinical value. Additionally, with the rise of drug use, abuse, and overdoses, early testing for addiction and other RDS subtypes is imperative. In the past, families would have never suspected that their loved ones may be in real danger due to an addiction or that they could potentially die. Author Bill Moyers reported in Parade Magazine that while traveling around the United States, he observed numerous children with ADHD and other spectrum disorders, such as autism. In addition, he noted that many of these children also suffered from related conditions, such as substance abuse. He urged for the development of more effective methods of identifying these children and alternative treatment options that did not involve addictive pharmaceuticals. In unpublished work, GARS was found to significantly correlate with the ASI-Media Version V alcohol and drug risk severity score [31]. While additional research is needed to confirm and extend the GARS test to include other genes and polymorphisms that are associated with hypodopaminergic traits, these findings provide clinicians with a non-invasive genetic test.

Genomic testing, such as GARS, can improve decision-making and clinical interactions [32]. Knowledge of precise polymorphic associations can aid in the attenuation of denial and guilt; corroboration of family gene-o-grams; assistance in risk-severity-based decisions about appropriate therapies, including pain medications and risk for addiction; choice of the appropriate level of care placement (i.e., outpatient, intensive outpatient, residential, inpatient); determination of treatment length of stay; determination of genetic severity-based relapse and recovery vulnerability and liability; determination of pharmacogenetic medical monitoring for improved clinical outcomes (i.e., the *A1 allele* of the *DRD2* gene decreases the binding to opioid delta receptors in the brain, thus diminishing Naltrexone’s clinical effectiveness [33]); and supporting medical necessity for insurance scrutiny.

Although not a magic bullet or “cure”, and despite the enormous efforts of the federal government to help fund, develop, and deliver treatments (MAT) to people with SUD, treatment penetration rates remain less than 20% [34]. McLellan et al. correctly pointed out that the diabetes field faced a similar dilemma [35]. By establishing the concept of “prediabetes”, early-stage diabetes detection was able to increase treatment penetration. In 2001, the American Diabetes Association suggested that the term “prediabetic” be operationally defined by augmented scores on two laboratory tests: impaired glucose tolerance and impaired fasting glucose [36]. This strategy led to a comprehensive campaign and partnership with third-party payors and, over time, has shown increased risk detection rates, shortened delays between symptom onset and treatment entry, and success in halting the progression of diabetes [37]. The emerging concept of “preaddiction” is thought-provoking, and, like “prediabetes”, if preaddiction is identified during the early stages of SUD, or better yet, before addictive behaviors manifest in an individual’s life, then it could also save lives. This may be the missing piece in solving drug abuse and addiction. The addiction field might characterize tests to explore preaddiction; for example, administering a RDS questionnaire (i.e., a 29-item Reward Deficiency Syndrome Questionnaire (RDSQ-29)) [38], a genetic risk assessment (i.e., GARS), a modified brain health check, or a diagnostic framing of mild to moderate SUD as pre-addiction could incentivize the development of interventions to prevent addiction from developing in the first place.

Lastly, many articles have discussed various options to treat and prevent all substance and non-substance addictive behaviors. Gold and associates and others have pioneered many of these clinically relevant options for an impressive list of substances, including marijuana, alcohol, opioids, psychostimulants, benzodiazepines, barbiturates, and process addictions [6,39,40,41,42,43,44,45]. One area of research that needs attention involves concepts related to negative emotionality [46].

## 5. Limitations

Our work has been based on a number of candidate gene methods, which were first initiated by the work of Blum and Noble in 1990 [10], as the first confirmed candidate gene to be associated with alcoholism, as well as a number of other classic candidate gene association studies in terms of accepted methodology [47,48,49,50]. While we are cognizant of a number of pitfalls related to the candidate gene approach, including ancestry, we believe the candidate approach currently has a clinically relevant outcome and heuristic value. Certainly, the psychiatric genetic field is moving towards GWAS instead of candidate gene research, but convergence to candidate genes is required to make real sense of the enormity of the data. One example of this type of GWAS analysis was conducted through a proxy-phenotype meta-analysis of problematic alcohol use disorder (PAU), combining alcohol use disorder and problematic drinking, in 435,563 individuals of European ancestry [51]. They identified 29 independent risk variants, 19 of them novel. PAU was genetically correlated with 138 phenotypes, including substance use and psychiatric traits. Phenome-wide polygenic risk score analysis in an independent biobank sample (BioVU, N = 67,589) confirmed the genetic correlations between PAU and substance use and psychiatric disorders.

Moreover, a GWAS study involving a sample size of 1.2 million individuals, involving both tobacco and alcoholism, discovered 566 genetic variants in 406 loci associated with multiple stages of tobacco use (initiation, heaviness, and cessation) as well as alcohol use, with 150 loci evidencing pleiotropic association [52]. However, when convergence was applied, the authors found evidence for the involvement of many systems in tobacco and alcohol use, including genes involved in dopaminergic, nicotinic, and glutamatergic neurotransmission. However, our concern related to these GWAS and our subsequent evaluation is that the controls, for the most part, utilized have not been adequately screened to eliminate all RDS symptomatology and associated disorders (i.e., gambling, hoarding, obesity, ADHD, etc.).

To help clarify and reiterate, these limitations may result from the investigators’ inadequate control screening for SUD and AUD, as well as any of the numerous RDS behaviors, such as obesity, nicotine dependence, pathological gambling, and internet gaming addiction [27]. The take-home message here is that unlike one-gene-one disease, RDS is polygenetic and extremely complex. In addition, any RDS-related behaviors must be removed from the control group in order to achieve the best statistical analysis and to prevent the phenotype from being compared to disease-ridden controls.

It is important to understand that the genes evaluated in this paper are not the only ones related to RDS. We focused on these specific genes and associated polymorphisms because the available GARS test only measures these genes selected on the basis of hypodopaminergia. Of course, other genes such as alcohol metabolism genes (i.e., alcohol dehydrogenase) combined with GARS may provide an even stronger association in terms of risk. Moreover, while it is true that there are many additional alleles that could help predict severe alcoholism/RDS, it is also true that the benefit of GARS is that it reflects the finite downstream major neurotransmitter systems rather than upstream, which could involve many other polymorphic alleles, including key second messengers, etc. It is also important to realize that the development of a useful test needs to be easy to interpret, which is another important benefit of employing a reductionist approach. It is of interest that these polymorphic SNPS are minor. However, just to be clear, for example, both *DRD4* and *DRD2* evolved in such a way that, in early species such as Neanderthals, both known polymorphisms were major, not minor. This is an adaptive trait potentially necessary for survival. While we found that at least in this nuclear family, knowing their GARS score provided some relief of personal guilt in terms of drug-seeking behavior and other unwanted behaviors, having a low GARS score may not resonate as strongly.

## 6. Summary

These results are encouraging and support our earlier work [28,30]. Our laboratory continues to perform research to effectively develop the Anti-RDS Modeling Solution System (ARDSMSS), consisting of a semi-customized precision KB220Z variant matched to the individuals’ GARS test result to treat the net dopamine dysfunction. This integrated “systems biology” approach provides an increase in efficacy in the treatment of RDS. Dopamine is a major neurotransmitter that is involved in substance and non-substance addictions. However, there is debate over the clinical management of dopamine in the prevention and treatment of many addictive disorders [53,54,55,56,57,58,59,60,61,62]. To assist the readership in comprehending specific effects of these various polymorphisms found in the family probands, we suggest reviewing our earlier paper published in 2014 in the Journal of Molecular Neurobiology [63].

## 7. Conclusions

**Figure 1:**
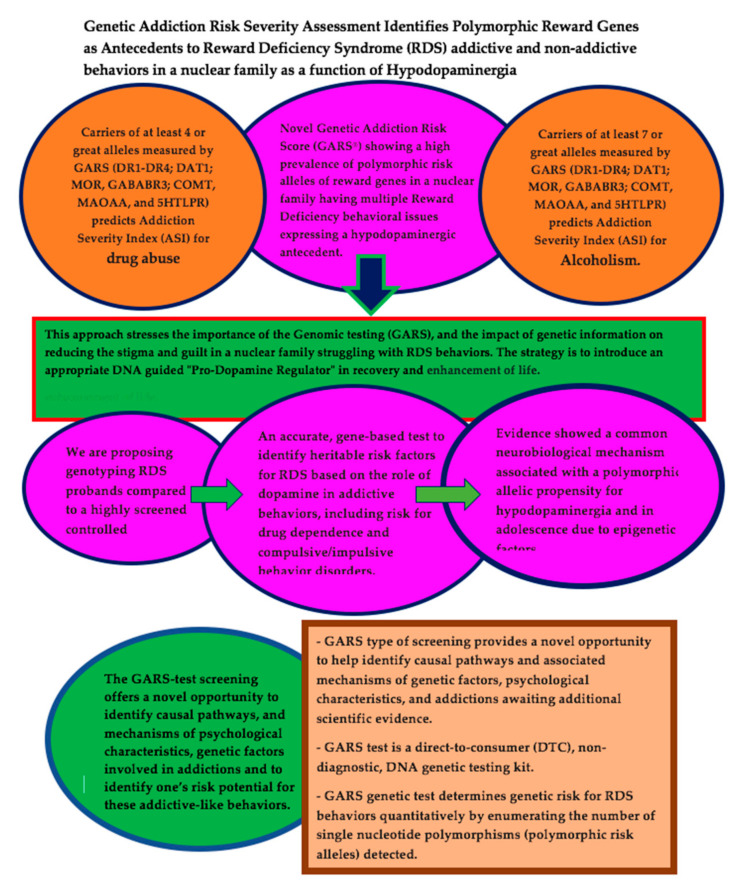
GARS assessment identifies polymorphic reward genes as antecedents to RDS addictive and non-addictive behaviors in a nuclear family as a function of hypodopaminergia.

This case series presents the novel GARS test, which shows a high prevalence of polymorphic risk alleles of reward genes in a nuclear family with multiple RDS behavioral issues expressing a hypodopaminergic antecedent (Figure 1). This generational case series represents an example of the impact of genetic information on reducing stigma and guilt in a nuclear family struggling with RDS behaviors. The futuristic plan is to introduce an appropriate DNA-guided “pro-dopamine regulator” into the recovery and enhancement of life.

## Figures and Tables

**Table 2:** Single nucleotide polymorphisms (SNPs) *.

Gene	Polymorphism	Variant Alleles	Risk Allele
*DRD1*	*rs4532*	A/G	A
*DRD2*	*rs1800497*	A/G (A1/A2)	A (A1)
*DRD3*	*rs6280*	C/T	C
*DRD4*	*rs1800955*	C/T	C
*COMT*	*rs4680*	A/G (Met/Val)	G (Val)
*OPRM1*	*rs1799971*	A/G (Asn/Asp)	G (Asp)

* Abbreviations: Dopamine receptor D1 (*DRD1*), dopamine receptor D2 (*DRD2*), dopamine receptor D3 (*DRD3*), dopamine receptor D4 (*DRD4*), catecholamine-methyltransferase (*COMT*), opioid receptor mu 1 (*OPRM1*).

**Table 3:** Simple Sequence Repeats (Variable Number Tandem Repeats and Insertion/Deletions) *.

Gene	Polymorphism	Variant Alleles	Risk Allele
*DRD4*	*rs761010487*	48bp repeat 2R-11R	≥7R, long form
*DAT1*	*rs28363170*	40p repeat 3R-11R	<9R
*MAOA*	*rs768062321*	30bp repeat 2R-5R	3.5R, 4R, 5R
*Serotonin Transporter SLC6A4* *(5-HTTLPR)*	*rs4795541*, *rs25531*	43bp repeat, with SNP L/XL and S, G/A	S, LG

* Abbreviations: Dopamine receptor D4 (*DRD4*), dopamine transporter (*DAT1*), monoamine oxidase A (*MAOA*), and serotonin-transporter-linked promoter region (*5HTTLPR*).

**Table 4:** Dinucleotide repeats *.

Gene	Polymorphism	Variant Alleles	Risk Allele
*GABA(A) Receptor*, *Alpha-3 GABRB3*	*rs764926719*	CA dinucleotide repeat 171–201bp sized fragments	181

* Abbreviations: Gamma-aminobutyric acid type A receptor subunit beta3 (*GABRB3*).

**Table 5:** Represents the GARS SNPS and VNTRs (snapshot) *.

Gene	Polymorphism	Location	Risk Allele(s)
*DRD1*	*rs4532* SNP	Chr 5	*A*
*DRD2*	*rs1800497* SNP	Chr 11	*A*
*DRD3*	*rs6280* SNP	Chr 3	*C*
*DRD4*	*rs1800955* SNP	Chr 11	*C*
	48 bases Repeat VNTR	Chr 11, Exon 3	*7R*, *8R*, *9R*, *10R*, *11R*
*COMT*	*rs4680* SNP	Chr 22	*G*
*OPRM1*	*rs1799971* SNP	Chr 6	*G*
*DAT1*	40 bases Repeat VNTR	Chr 5, Exon 15	*3R*, *4R*, *5R*, *6R*, *7R*, *8R*
*MAOA*	30 bases Repeat VNTR	Chr X, Promoter	*3.5R*, *4R*
*SLC6A4 (5HTTLPR)*	43 bases Repeat INDEL/VNTR plus *rs25531* SNP	Chr 17	*LG*, *S*
*GABA(A) Receptor*, *Alpha-3 GABRB3*	CA-Repeat DNR	Chr15 (downstream)	*181*

* Abbreviations: Dopamine receptor D1 (*DRD1*), dopamine receptor D2 (*DRD2*), dopamine receptor D3 (*DRD3*), dopamine receptor D4 (*DRD4*), catecholamine-methyltransferase (*COMT*), opioid receptor mu 1 (*OPRM1*), dopamine transporter (*DAT1*), monoamine oxidase A (*MAOA*), and serotonin-transporter-linked promoter region (*5HTTLPR*), gamma-aminobutyric acid type A receptor subunit beta3 (*GABRB3*), single nucleotide polymorphism (SNP), variable number tandem repeat (VNTR).

**Table 6:** Global heterozygous prevalence.

SNP	Global Heterozygous Prevalence
*rs4532*	32%
*rs1800497*	46%
*rs6280*	41%
*rs1800955*	Frequency of C allele = 0.42 Prevalence not available
*rs4680*	42%
*rs1799971*	29%

**Table 7:** GARS repeat primer details.

Primer	Sequence (5′ to 3′)	5′ Label	Reaction (nM)
AMELO-F AMELO-R	CCC TGG GCT CTG TAA AGA ATA GTG ATC AGA GCT TAA ACT GGG AAG CTG	NED -	150
MAO-F MAO-R	ACA GCC TGA CCG TGG AGA AG GAA CGG ACG CTC CAT TCG GA	NED -	120
DAT-F DAT-R	TGT GGT GTA GGG AAC GGC CTG AG CTT CCT GGA GGT CAC GGC TCA AGG	6FAM -	120
DRD4-F DRD4-R	GCT CAT GCT GCT GCT CTA CTG GGC CTG CGG GTC TGC GGT GGA GTC TGG	VIC -	480
GABRA-F GABRA-R	CTC TTG TTC CTG TTG CTT TCA ATA CAC CAC TGT GCT AGT AGA TTC AGC TC	NED -	120
HTTLPR-F HTTLPR-R	ATG CCA GCA CCT AAC CCC TAA TGT GAG GGA CTG AGC TGG ACA ACC AC	PET -	120

**Table 8:** GARS single nucleotide polymorphism assay information—TaqMan *.

Assay ID	Gene and SNP	Context Sequence
C 1011777_10	*DRD1 rs4532*	TCTGACTGACCCCTATTCCCTGCTT [G/A] GGAACTTGAGGGGTGTCAGAGCCCC
C 7486676_10	*DRD2, ANKK1 rs1800497*	CACAGCCATCCTCAAAGTGCTGGTC [A/G] AGGCAGGCGCCCAGCTGGACGTCCA
C 949770_10	*DRD3 rs6280*	GCCCCACAGGTGTAGTTCAGGTGGC [C/T] ACTCAGCTGGCTCAGAGATGCCATA
C 7470700_30	*DRD4 rs1800955*	GGGCAGGGGGAGCGGGCGTGGAGGG [C/T] GCGCACGAGGTCGAGGCGAGTCCGC
C 25746809_50	*COMT rs4680*	CCAGCGGATGGTGGATTTCGCTGGC [A/G] TGAAGGACAAGGTGTGCATGCCTGA
C 8950074_1_	*OPRM1 rs1799971*	GGTCAACTTGTCCCACTTAGATGGC [A/G] ACCTGTCCGACCCATGCGGTCCGAA

* Abbreviations: Dopamine receptor D1 (*DRD1*), dopamine receptor D2 (*DRD2*), dopamine receptor D3 (*DRD3*), dopamine receptor D4 (*DRD4*), catecholamine-methyltransferase (*COMT*), opioid receptor mu 1 (*OPRM1*).

**Table 13:** Summary of family RDS behavior (self-reported but backed previously diagnosed by a physician).

Proband	RDS Behavior (s) and Psychiatric Conditions
Mother	Gaming Memory Focus Addicted to TV shows Anxiety ADHD
Father	Overeating ADHD
Son	Substances-misuse Cannabis and Alcohol Sweets Depression Anxiety ADHD Amotivation Risk Taking
Daughter	Internet-misuse Depression Anxiety

## Data Availability

Data are available within the manuscript.
